# Increased Suitability of Poleward Climate for a Tropical Butterfly (*Euripus nyctelius*) (Lepidoptera: Nymphalidae) Accompanies its Successful Range Expansion

**DOI:** 10.1093/jisesa/iez105

**Published:** 2019-11-08

**Authors:** Tsun Fung Au, Timothy C Bonebrake

**Affiliations:** 1 School of Biological Sciences, Faculty of Science, The University of Hong Kong, Hong Kong, China; 2 Department of Geography, Faculty of Social Science, The University of Hong Kong, Hong Kong, China; 3 Department of Geography, Indiana University, Bloomington, IN

**Keywords:** butterflies, climate change, species redistribution, species distribution model, thermal tolerance

## Abstract

Distribution shifts are a common response in butterflies to a warming climate. Hong Kong has documented records of several new butterfly species in recent decades, comprising a high proportion of tropical species, some of which have successfully established. In this study, we examined possible drivers for the establishment of *Euripus nyctelius* Doubleday (Lepidoptera: Nymphalidae) by studying its thermal physiology and modeling current climate and future distributions projected by species distribution modeling (SDM). We found that *E. nyctelius* adults have a significantly higher critical thermal minimum than its local temperate relative, *Hestina assimilis* Linnaeus (Lepidoptera: Nymphalidae), suggesting a possible physiological constraint that may have been lifted with recent warming. SDMs provide further evidence that a shifting climate envelope may have improved the climate suitability for *E. nyctelius* in Hong Kong and South China—however, we cannot rule out the role of other drivers potentially influencing or driving range expansion, habitat change in particular. Conclusive attribution of warming-driven impacts for most tropical species is difficult or not possible due to a lack of historical or long-term data. Tropical insects will require a significant advancement in efforts to monitor species and populations across countries if we are to conclusively document climate-driven shifts in species distributions and manage the consequences of such species redistribution. Nevertheless, the warming climate and subsequent increased climatic suitability for tropical species in poleward areas, as shown here, is likely to result in future species redistribution events in subtropical and temperate ecosystems.

Anthropogenic greenhouse gas emissions have increased surface temperature by 0.85°C globally over the past century and the global mean temperature is projected to increase 1.5 to 2°C by the end of the 21st century (IPCC 2013). This warming has already caused significant disruption to ecosystems and associated fauna and flora (Scheffers et al. 2016). One common response to climate change is species redistribution across elevation and latitude, with such distribution shifts occurring widely and globally (Hickling et al. 2006, Pecl et al. 2017). Latitudinal shifts in butterflies are well documented in the Northern Hemisphere, especially temperate regions (Parmesan 1996). For example, among 35 European butterflies examined, 22 extended their distributions northwards by 35–240 km during the 20th century (Parmesan et al. 1999). Similarly, in the northeast United States, 17 out of 21 butterfly species experienced southern range declines following warming (Breed et al. 2013).

Tropical regions have the highest biodiversity in the world but climate change impacts on tropical butterflies are largely undocumented (Bonebrake et al. 2010). In tropical Asia, moths have exhibited elevation shifts in recent decades consistent with climate change (Chen et al. 2009, Cheng et al. 2019). Range expansion of butterflies are also evident in tropical Asia. For example, several species with unclear origins have recently arrived on Lombok (Indonesia) (Matsumoto et al. 2012). In examining possible causes behind the Australian colonization of a southeast Asia butterfly species *Acraea terpsicor*, Braby et al. (2014) attributed its distribution changes to rapid deforestation in southeast Asia driving range expansion into Australia and, using bioclimatic niche models, future warming was also projected to facilitate its spread. Overall, whether such butterfly range shifts in tropical Asia are driven by habitat modification, extreme events, or climate change remain unknown.

Hong Kong has a subtropical monsoon climate characterized by rich biodiversity (Dudgeon and Corlett 2004), with over 230 species of butterflies recorded as resident (Lo and Hui 2004, Chan et al. 2011). Several butterfly and moth species have become newly established over the past two decades and climate change has been proposed as a potential driving force behind these colonizations (Chan et al. 2011, Cheng et al. 2019). Some newly established butterflies are of tropical origin, for example, *Euripus nyctelius* Doubleday (Lepidoptera: Nymphalidae) and *Lexias pardalis* (Moore, 1878) (Lepidoptera: Nymphalidae), first discovered in Hong Kong in 2007 and 2008, respectively. The establishment of these species in Hong Kong could be a consequence of higher winter minimums or higher summer maximums facilitating their survival in these novel habitats. To test this hypothesis, we compared thermal tolerance limits of the newly established *E. nyctelius* and one of its local relatives, *Hestina assimilis* Linnaeus (Lepidoptera: Nymphalidae). The subtropical and temperate origin of *H. assimilis* and the tropical origin of *E. nyctelius* allows ecophysiological comparison such that we can test the hypothesis that the tropical species *(E. nyctelius*) should have narrower thermal tolerance breadth than the temperate species *(H. assimilis*) (Deutsch et al. 2008, Bonebrake et al. 2014). We additionally employed species distribution models (SDMs) to quantify changing climatic suitability for these species over time.

## Materials and Methods

### Study Species

The target species of this study is *E. nyctelius* (Doubleday, 1848) (The Courtesan), a Nymphalidae species first recorded in Hong Kong in 2007 (Chan et al. 2011). *E. nyctelius* is found in southern China, including Guangxi, Hainan, and southern Yunnan but is also widely distributed in Vietnam, Laos, Cambodia, Thailand, Malaysia, Singapore, Philippines, and north-eastern India (Corbet and Pendlebury 1992, Khew 2010, Ek-Amnuay 2012, Lang 2012) (Supp Fig. 1 [online only]). Adult *E. nyctelius* is characterized by its yellowish eyes but adults also exhibit a sexual dimorphism. Males are generally smaller than females and have white spots and patches on both the upperside and underside of wings. Females display Batesian mimicry with the genus *Euploea* (Chou 1994) and share similarities to *Euploea midamus*, *Euploea core*, and *Euploea mulciber* in Hong Kong. The undersides of both forewings and hindwings are brownish with white spots. The forewing upperside has a metallic blue color under strong light and the hindwing is brownish. Local observations of *E. nyctelius* are typically from forested areas. The larval host plant is *Trema tomentosa* (Roxb.) Hara (Rosales: Cannabaceae) (Young et al. 2008), a common native tree species in Hong Kong, widely distributed in tropical and subtropical Asia (Hu and Wu 2007). Overwintering and diapause are not known for this species.

A control species was selected as a means of comparison for physiological characteristics with *E. nyctelius*. The purpose for the control species was to set up a baseline to reveal the physiological limits that might be suitable to both past and current climate conditions in Hong Kong. In order to minimize distinct interspecific disparities, three selection criteria were established for species choice: 1) the species must be closely related to *E. nyctelius*; 2) the species should share morphological similarities with *E. nyctelius* in both adult and larval forms; and 3) the species must be a native species that persisted in Hong Kong such that physiological limits are unlikely to have changed significantly.

We selected *Hestina assimilis* as the best match to these criteria (Linnaeus, 1758) (Red Ring Skirt), a native Hong Kong species recorded in an early checklist published in 1893 (Skertchly and Walker 1893). *H. assimilis* is phylogenetically clustered with *E. nyctelius* based on mitochondrial DNA analysis (Zhang et al. 2007) and morphologically similar to *E. nyctelius* (Zhang et al. 2008) under the same subfamily Apaturinae. *H. assimilis* is common in Hong Kong and widely distributed in East Asia and China, including Guangdong, Guangxi, Sichuan, Guizhou, Jiangxi, Shanghai, Hebei, Beijing, Heilongjiang, as well as in Korea and Japan (*H. assimilis* was introduced to Japan in the 1990s and is non-native there) (Lang 2012, Karasawa et al. 2012). Adults appear in two forms: normal form *H. assimilis assimilis* occurs year-round while the pale form *H. assimilis nigrivena* is only found during the spring (Lo and Hui 2004). *H. assimilis* usually inhabits secondary forests but can be found occasionally in urban parks if its known host plant *Celtis sinensis* Pers. (Rosales: Cannabaceae) is present (Bascombe et al. 1999). *H. assimilis* overwinters mainly in larval form (Bascombe et al. 1999) with a brownish exoskeleton.

### Collection Sites

Larvae of *E. nyctelius* and *H. assimilis* were collected in different locations across Hong Kong where sufficient numbers of individuals could be found. *E. nyctelius* larvae were only collected in Sai Kung because of easy accessibility to a well-known established local population. *H. assimilis* larvae were collected in different parts of Hong Kong, including the New Territories and Hong Kong Island. The collection period was from June to September 2016.

### Thermal Tolerance

Extreme temperatures such as minimum and absolute minimum temperatures play a critical role in determining species performance, survival, and distributions (Sheldon and Dillon 2016). Therefore, for both *E. nyctelius* and *H. assimilis*, we measured critical thermal minima (CT_min_) for both larva and adult stages and critical thermal maxima (CT_max_) for adults only. After field sampling, the larvae and adults were kept and reared in temperature-controlled chambers (Panasonic MIR-254-PE) at 22°C at the University of Hong Kong. Each larva was separated by an individual plastic container to avoid potential cross-contamination and disease. The larvae were provided with their corresponding freshly cut host plant leaves collected at Lung Fu Shan Country Park. Frass was cleaned every day and containers sterilized with 95% ethanol at least once a week to assure healthy growth.

When larvae reached the fifth instar and within 3 d of the time adults emerged from pupae, we measured body weight and measured CT_min_. For larval CT_min_ measurements, we followed the protocol of Klok and Chown (1997), one of the few studies testing Lepidoptera larval CT_min_. Klok and Chown (1997) defined the temperature that the larva regained mobility as CT_min_ for that individual, or the recovery temperature from chill coma (Terblanche et al. 2011). We found this protocol appropriate for our experiment because observation of mobility is clearer and more easily detected than observation of immobility, especially in almost immobile butterfly caterpillars or a resting butterfly adult. For larval CT_min_ measurements, environmental chambers were precooled to 4.5 ± 0.5°C. Then, the test subjects were put in the chamber for 15 min to reach equilibrium at which point the subjects were inactive. After 15 min, the temperature was gradually heated up to 22°C with a ramping rate of around 0.5°C per minute to ensure the larvae experienced the same temperature as the chamber. For adult CT_min_ measurements, the same procedure was applied, except one extra step was added after the subjects became inactive. We adopted the step described by Andrew and Kemp (2016): adult butterflies clinging to the sides of the transparent plastic box were removed by tapping the box gently to assure all subjects lay flat at the bottom to facilitate detection of mobility.

For CT_max_ measurement, only adult individuals were assessed because previous studies have shown exposure to high temperatures can cause detrimental, even lethal impacts for larval development (Zalucki 1982, Malcolm et al. 1987). After adult CT_min_ assays, individuals were kept in the chamber for 3 d at 22°C to allow stabilization before CT_max_ measurement. Similar to the CT_min_ measurement, we used environmental chambers with temperature progressively increased from 22°C with ramping rate of 0.5°C per minute until individuals lost motor control. Given the relatively slow ramping rate in both CT_min_ and CT_max_ measurements, we assumed larval and adult body temperatures were the same as the air temperature of the chamber, and so we inferred organismal body temperature directly to be the air temperature of the environmental chamber.

### Data Analysis

We used CT_min_ and a standardized CT_min_ value taken by dividing the weight of corresponding larvae or adults; body size may impact thermal resistance, especially cold tolerance (Huey et al. 1992, Heinze et al. 1998). We employed two-sample *t*-tests to analyze the inter-specific differences of CT_min_ between *E. nyctelius* and *H. assimilis* for larval stage and adult stages, respectively. We also employed paired sample *t*-tests to analyze intra-specific differences of CT_min_ between larval and adult thermal tolerance for the same individual.

We calculated the thermal suitability for both species by taking the minimum winter (January and February) temperature of Hong Kong. We first averaged January and February monthly minimum temperature (T_min_) since 1947, obtained from the Hong Kong Observatory (http://www.hko.gov.hk/cis/monthlyExtract_e.htm). The average yearly winter T_min_ was then subtracted by larval and adult mean CT_min_ of both species from the year of 1947 to 2017. The difference between winter T_min_ and mean CT_min_ provides a ‘thermal safety margin’ (Deutsch et al. 2008) for both species at corresponding life stages.

### Species Distribution Modeling

Distribution data were obtained from a variety of sources, including books, the scientific literature, and internet records or databases. We adopted the same approach as Xing et al. 2019 in searching distribution records for both *E. nyctelius* and *H. assimilis.* For the scientific literature and internet records published online, we typed in ‘Euripus’ and ‘Hestina’ to avoid missing the species synonyms used in the past as well as their common names in Chinese (‘芒蛺蝶’ for *E. nyctelius* and ‘黑脈蛺蝶’ for *H. assimilis*) for searching past occurrences on google scholar, google and the Global Biodiversity Information Facility (GBIF). The search for occurrence records yielded 106 and 69 points for *E. nyctelius* and *H. assimilis*, respectively, for spatial analysis with the oldest observation record in 1887 (Supp Fig. 1 and Supp Table 1 [online only]). We compiled these records from September to December 2016.

For bioclimatic niche modeling, we obtained 19 bioclimatic variables at 2.5 min spatial resolution (~4.65 × 4.65 km at the equator) from WorldClim version 1.4 (Hijmans et al. 2005) and examined collinearity of the 19 bioclimatic variables using the *vif* function in the *usdm* package (R v1.1–15, Naimi et al. 2014). We eventually identified and used nine bioclimatic variables with *vif* <10 to perform niche modeling (Supp Table 2 [online only]). We used eight modeling techniques provided by *biomod2* package (R.v.3.3–7, Thuiller et al. 2016) in R (R core team 2014), namely, artificial neural networks (ANN), classification tree analysis (CTA), multiple adaptive regression splines (MARS), surface range envelope (SRE), random forest (RF), generalized boosted models (GBM), generalized linear models (GLMs), and flexible discriminant analysis (FDA) (Guisan et al. 2017, Xing et al. 2019). Both presence and pseudo-absence data have the same weight for distribution modeling. We employed 70% of the locality data for model calibration and the remaining 30% for model evaluation. We evaluated the prediction of the eight models over three pseudo-absence selections with 10 cross-validation runs operated by the true skill statistic (TSS) and relative operating characteristic (ROC) (Guisan et al. 2017). For future bioclimate data, two greenhouse gas concentration scenarios RCP2.6 and RCP8.5 in the year of 2050 were calibrated from the Fifth IPCC assessment report (IPCC 2014). We used three global circulation models (GCMs): HadGEM2-AO (Bellouin et al. 2011), IPSL-CM5A-LR (Dufresne et al. 2013), and MRI-CGCM3 (Yukimoto et al. 2012) to perform future distribution modeling. The final ensemble distribution suitability maps are based on the models with a TSS over 0.8 and area of curve (AUC) of ROC over 0.7 to project habitat suitability for both species (Guisan et al. 2017, Xing et al. 2019). Changes in habitat suitability were calculated by the differences between the future GCMs and predicted current distributions. The habitat suitability changes of all three GCMs were then averaged to present consensus prediction on habitat suitability under both emissions scenarios in 2050. The variable importance values of nine bioclimatic variables were also calculated by averaging the importance values generated by the eight modeling techniques employed from 30 runs in total.

## Results

In total, 29 *E. nyctelius* individuals (3 eggs, 25 larvae, and 1 adult) and 20 *H. assimilis* individuals (3 eggs and 17 larvae) were collected during the sampling period. Fourteen *E. nyctelius* and 12 *H. assimilis* adult individuals grew healthy and successfully emerged from pupae. Three adult individuals of each species were prepared for pilot experiments in order to finalize experimental procedures and, therefore, were not included in data analysis.

### Larval and Adult CT_min_ and Adult CT_max_

**Fig. 1.**
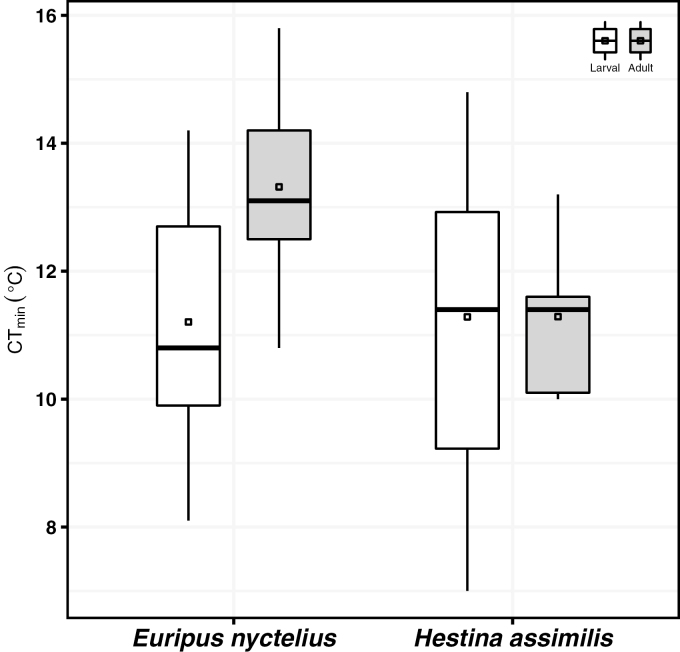
Larval (white) and adult (gray) critical thermal minima (CT_min_) of *Euripus nyctelius* and *Hestina assimilis* in Hong Kong.

The mean CT_min_ of *E. nyctelius* (*n* = 17) and *H. assimilis* (*n* = 14) larvae were 11.21 and 11.29°C, respectively (Fig. 1). For standardized larval CT_min_, *E. nyctelius* (*n* = 10) and *H. assimilis* (*n* = 12) larvae regained activity at 12.21°C/g and 12.42°C/g, respectively. Both normal (*t* = -0.104, df = 29, *P* = 0.918) and standardized (*t* = -0.093, df = 20, *P* = 0.927) CT_min_ showed no significant differences between *E. nyctelius* and *H. assimilis* larvae. The mean adult CT_min_ of *E. nyctelius* (*n* = 11) and *H. assimilis* (*n* = 9) was 13.32 and 11.29°C, respectively (Fig. 1). Adult CT_min_ was significantly higher for *E. nyctelius* relative to *H. assimilis* (*t* = 3.40, df = 18, *P* = 0.003) (Fig. 1). Among these individuals examined, eight *E. nyctelius* and six *H. assimilis* individuals survived in both larval and adult stages. The CT_min_ of these eight *E. nyctelius* larvae and adults were 11.14 and 13.59°C, respectively, showing a significant 2.45°C increase in CT_min_ from larval stage to adult stage (*t* = -3.818, df = 7, *P* = 0.007) (Supp Fig. 2 [online only]). The CT_min_ of larval and adult stages were 10.75 and 11.53°C, respectively for these six *H. assimilis* individuals but no significant disparity was found between larval and adult stages (*t* = -0.732, df = 5, *P* = 0.497) (Supp Fig. 2 [online only]). The adult CT_max_ of *E. nyctelius* (*n* = 8) and *H. assimilis* (*n* = 4) was 43.39 and 44.08°C, respectively. No significant difference was found between *E. nyctelius* and *H. assimilis* CT_max_ (*t* = -0.56, df = 10, *P* = 0.588) but the sample size was very low, limiting inference (see discussion below).

### Thermal Safety Margin

**Fig. 2.**
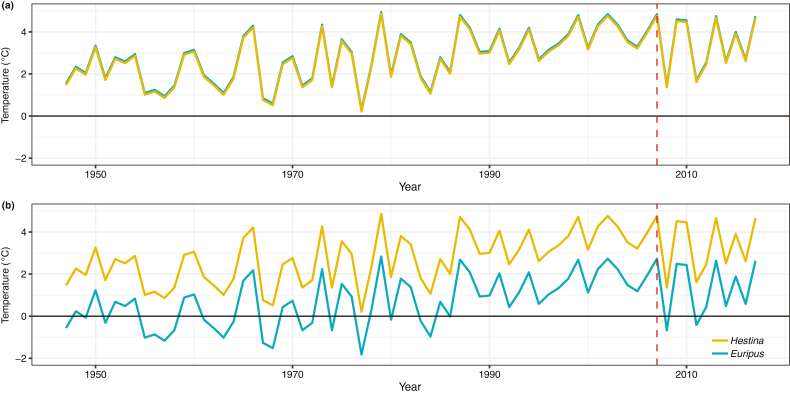
Thermal safety margins of *Euripus nyctelius* (blue) and *Hestina assimilis* (yellow) during their (a) larval stage and (b) adult stages since 1947. Positive values above zero suggest suitability to Hong Kong’s climate conditions. The vertical red line indicates the year (2007) when *Euripus nyctelius* was first recorded in Hong Kong.

The suitability of both species to winter temperatures increased over time. We found that larval suitability for both species is equal over time due to their similar mean CT_min_ (Fig. 2a). For adults, however, *H. assimilis* has a higher tolerance to winter temperatures as a consequence of the lower CT_min_. *E. nyctelius* adults have had increased suitability in Hong Kong with increasing air temperature since the 1980s (Fig. 2b).

### Species Distribution Models

**Fig. 3.**
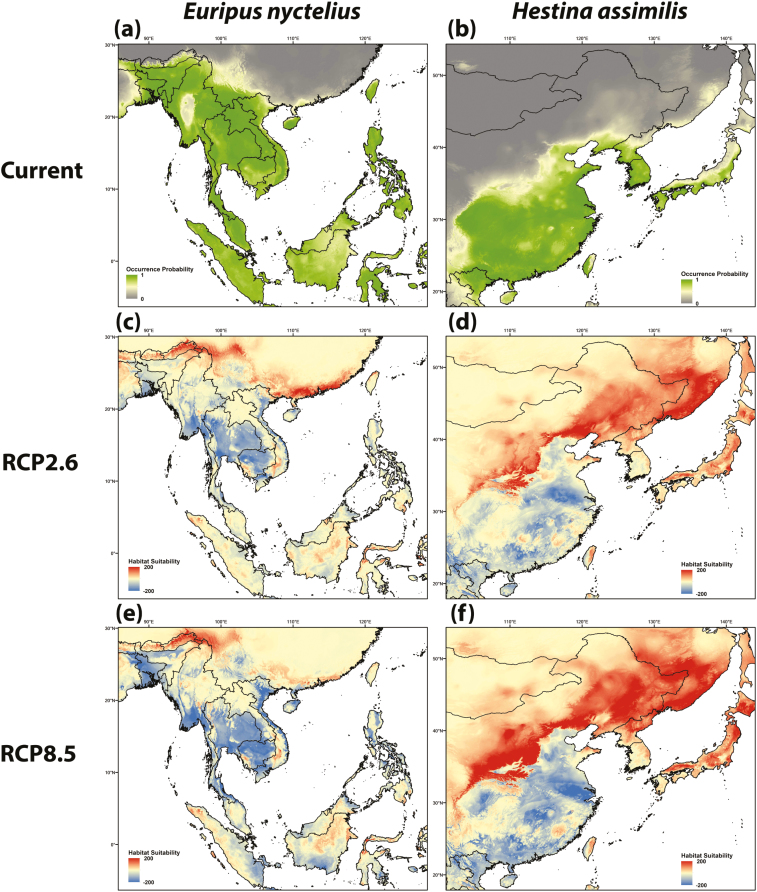
Current projected distribution of (a) *Euripus nyctelius* and (b) *Hestina assimilis* by ensembles of species distribution models. Green indicates higher probability for the species while the areas with dark gray indicate lower probability of occurrence. Projections of *E. nyctelius* and *H. assimilis* distributions under future emissions scenarios (c and d) RCP2.6 and (e and f) RCP8.5 in 2050 by are an average of three global circulation models (GCMs): HadGEM2-AO, IPSL-CM5A-LR, and MRI-CGCM3. Red indicates higher suitability while blue indicates reduced suitability relative to projected current distributions.

The SDMs generated under current climate conditions (Fig. 3) showed similar distributions for both species as observed from the scientific literature and guides (Supp Fig. 1 [online only]). The future distribution of *E. nyctelius* has the potential to be extended in southern China for RCP2.6 but such expansion is weakened in the RCP8.5 projection. Both scenarios show projections of retreating southern ranges of *E. nyctelius* in 2050. For *H. assimilis*, the projected northward latitudinal shift extent is stronger than *E. nyctelius* while reduction of southern habitat suitability was also projected for both RCP2.6 and RCP8.5 scenarios in 2050. Among all nine bioclimate variables used in computing SDMs, temperature seasonality (BIO4) had the highest importance value (0.668) in determining *E. nyctelius* distribution while precipitation of the warmest quarter (BIO18) plays a more important role (0.371) in the *H. assimilis* distribution.

## Discussion

The higher critical thermal minimum (CT_min_) found in *E. nyctelius* adults relative to *H. assimilis* points to the possibility that lower thermal limits may have prevented their establishment in Hong Kong historically. The positive thermal safety margin found (mean air temperature above CT_min_) for *E. nyctelius* only in recent decades (contrasting with *H. assimilis* whose thermal safety margin has historically always been positive) provides further evidence of potential climatic limits to its distribution. Finally, trends in climatic suitability as indicated by species distribution modeling corroborate each of these findings. Taken altogether these results are consistent with claims that climatic warming likely contributes to butterfly species redistribution in the Asian tropics, and in Hong Kong specifically.

Butterfly responses to climate change will be a consequence of the thermal biology of each life stage. For example, Kingsolver et al. (2011) found that *Colias* (Lepidoptera: Pieridae) larvae do not actively thermoregulate but lower temperature favors food ingestion and growth while adults require high internal body temperatures of 35–38°C for active flight (Watt 1968) crucial for reproduction. In order to achieve desired thoracic temperature and adapt to local climate, adult butterflies can adjust their body temperature by behavioral responses (e.g., basking) (Bonebrake et al. 2014) and morphological traits (e.g., wing melanin and setae on the ventral thorax) (Kingsolver 1983, 2011). The differences in physiological traits between *E. nyctelius* larvae and adults may be an indication of variable sensitivities across life stages. For *H. assimilis*, although larvae and adults do not exhibit a significant difference in CT_min_, larvae undergo a winter diapause (Bascombe et al. 1999) by reducing prothoracicotropic hormone and ecdysteroid hormones production to prevent molting (Denlinger et al. 2012, Nylin 2013). Further investigation of the developmental and thermal biology of each species would help to reveal the mechanisms that might limit *E. nyctelius* and *H. assimilis* distributions.

We propose three not mutually exclusive explanations for the recent establishment of *E. nyctelius* in Hong Kong: 1) *E. nyctelius* has existed in Hong Kong for a long time but was misidentified with other similar species, 2) *E. nyctelius* was accidentally introduced by humans or with its specific host plants, and 3) *E. nyctelius* was attracted by favorable climate conditions and habitats. Unlike species from small butterfly families (Lycaenidae and Hesperiidae), *E. nyctelius* belongs to Nymphalidae and is unlikely to be misidentified. The distinctive yellowish eyes of both males and females enable most lepidopterists to differentiate them from *H. assimilis* and other mimics. *E. nyctelius* is also unlikely to have been manually introduced. Hong Kong has documented numerous vagrant butterfly species (Lo and Hui 2004, Chan et al. 2011) but almost none of them successfully established in Hong Kong even when suitable host plants were readily available. For example, Lo and Hui (2004) believed that *Chilades pandava* larvae (Lepidoptera: Lycaenidae), a recent successful establishment in Hong Kong, accompanied its host plant *Cycas* to Hong Kong as Hong Kong does not have native *Cycas* and *Cycas* are often imported for horticulture purposes. In contrast, there is no evidence showing large-scaled importation of *Trema tomentosa*, the host plant of *E. nyctelius*. Furthermore, *Trema tomentosa* is already a native and readily available tree species in Hong Kong (Hu and Wu 2007).

With climatic warming, *E. nyctelius* thermal suitability has increased over time and, based on our CT_min_ results, may have made colonization in Hong Kong possible in the 1980s (Fig. 2b). The establishment of the species in 2007 (Chan et al. 2011) suggests that other factors were important in allowing establishment or that dispersal took time for the species to arrive. Our SDM projections for *E. nyctelius* show northern range expansion into South China, including Hong Kong in RCP2.6 (Fig. 3c) and a more extensive retreat in its southern distribution in RCP8.5, including Myanmar, Thailand, and Cambodia (Fig. 3e). These results validate the hypothesis that warming has improved the suitability of Hong Kong’s climate for *E. nyctelius* and could have influenced its establishment. But the projections also suggest that continued warming might influence further expansion and potentially result in decline at warm range edges (Wiens 2016). Climate change could have significant consequences for tropical butterflies and conservation interventions in Hong Kong and tropical Asia more broadly are needed to manage these impacts (Cheng and Bonebrake 2017).

Land-use change has had and will continue to have a primary role in driving species redistribution (Guo et al. 2018). For example, tropical rainforests in Southeast Asia are experiencing high deforestation rates (Achard et al. 2002, Miettinen et al. 2011) and habitat modification by deforestation has been proposed as a driver for range expansion in butterflies (Braby et al. 2014). Regarding *E. nyctelius*, the closest known locality to Hong Kong, Wuzhishan, Hainan Island reported by Lang (2012), has suffered from high rates of land-use change from natural forest to pulp and rubber plantation in the Changhua watershed (including parts of Wuzhishan) (Zhai et al. 2012, 2015). In contrast, Hong Kong has undergone considerable afforestation for erosion control since the 1870s with a focus on planting native floral species in recent decades to improve ecological value and facilitate natural forest regeneration (Corlett 1999). This has likely increased the habitat suitability for forest-associated *E. nyctelius*. Such habitat degradation in Southeast Asia and ecological restoration in Hong Kong may result in a push–pull model, facilitating *E. nyctelius* establishment. Urbanization has also been demonstrated to alter insect communities in complex ways (Tsang and Bonebrake 2017, Merckx et al. 2018) and can enhance larval survival and fitness for some species as a consequence of higher temperatures in urban settings (Kaiser et al. 2016). Although *E. nyctelius* prefers forested areas in Hong Kong, urbanization does play an important role in butterfly colonization in the region. For example, *Chilades pandava* expanded its distribution into Taiwan through non-native host plant introduction in urban areas (Wu et al. 2010)—the species likely established itself in Hong Kong similarly as discussed above (Lo and Hui 2004).

Tropical insects are experiencing significant declines as a consequence of complex changes in climate (Janzen and Hallwachs 2019). Maintenance of proper landscapes with high connectivity and heterogeneity play critical roles in helping species persist in the face of such global change (Koh and Sodhi 2004). We recommend further tropical butterfly monitoring at species and population levels (Bonebrake et al. 2010) with higher resolution of distribution data and the formulation of conservation strategies to manage distribution shifts across countries (Bonebrake et al. 2018, Xing et al. 2019).

## Supplementary Material

iez105_suppl_Supplementary_Figures_and_Tables

iez105_suppl_Supplementary_Table-S1
