# Supplementary material for: Increased Suitability of Poleward Climate for a Tropical Butterfly (Euripus nyctelius) (Lepidoptera: Nymphalidae) Accompanies its Successful Range Expansion
Source: J Insect Sci. 2019 Nov 8;19(6):2. doi: 10.1093/jisesa/iez105 (PMC6839647; doi:10.1093/jisesa/iez105)
Supplement: iez105_suppl_Supplementary_Figures_and_Tables [file iez105_suppl_supplementary_figures_and_tables.docx]

**Supplementary Figures and Tables**


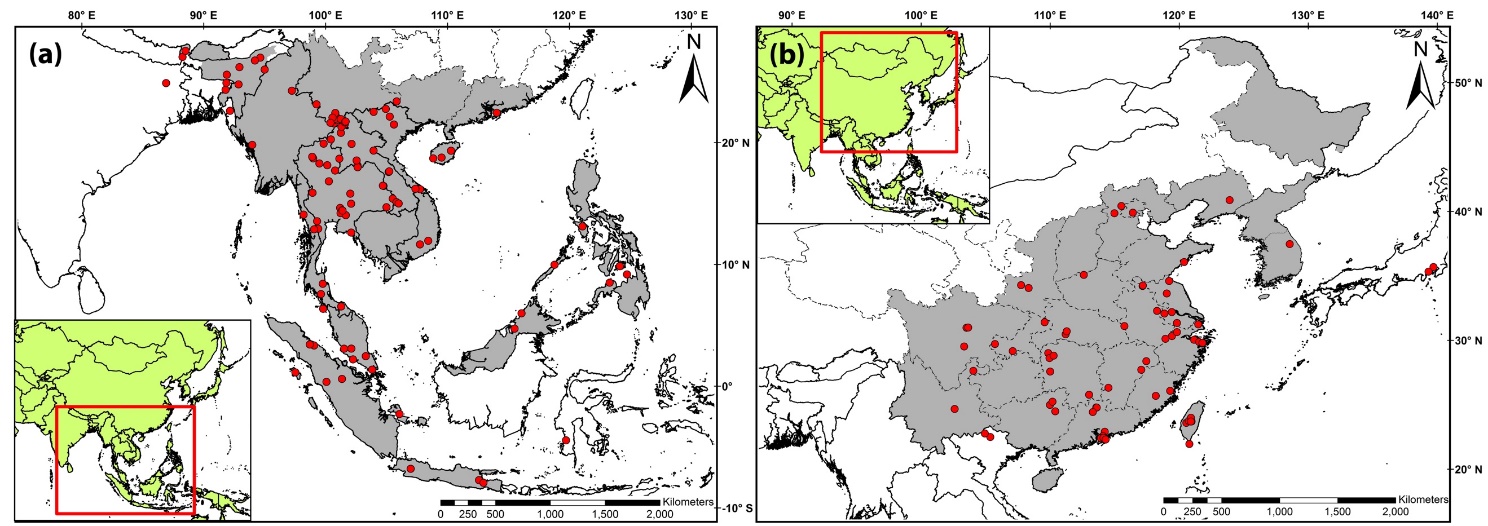


**Supp. Figure S1.** Spatial distribution of (a) *Euripus nyctelius* and (b) *Hestina assimilis*. Red dots indicate past and current occurrence data obtained from various literature and online sources (Appendix S1). Grey areas indicate recent ranges reported from *The Nymphalidae of China (Lepidoptera, Rhopalocera)* (Lang 2012).


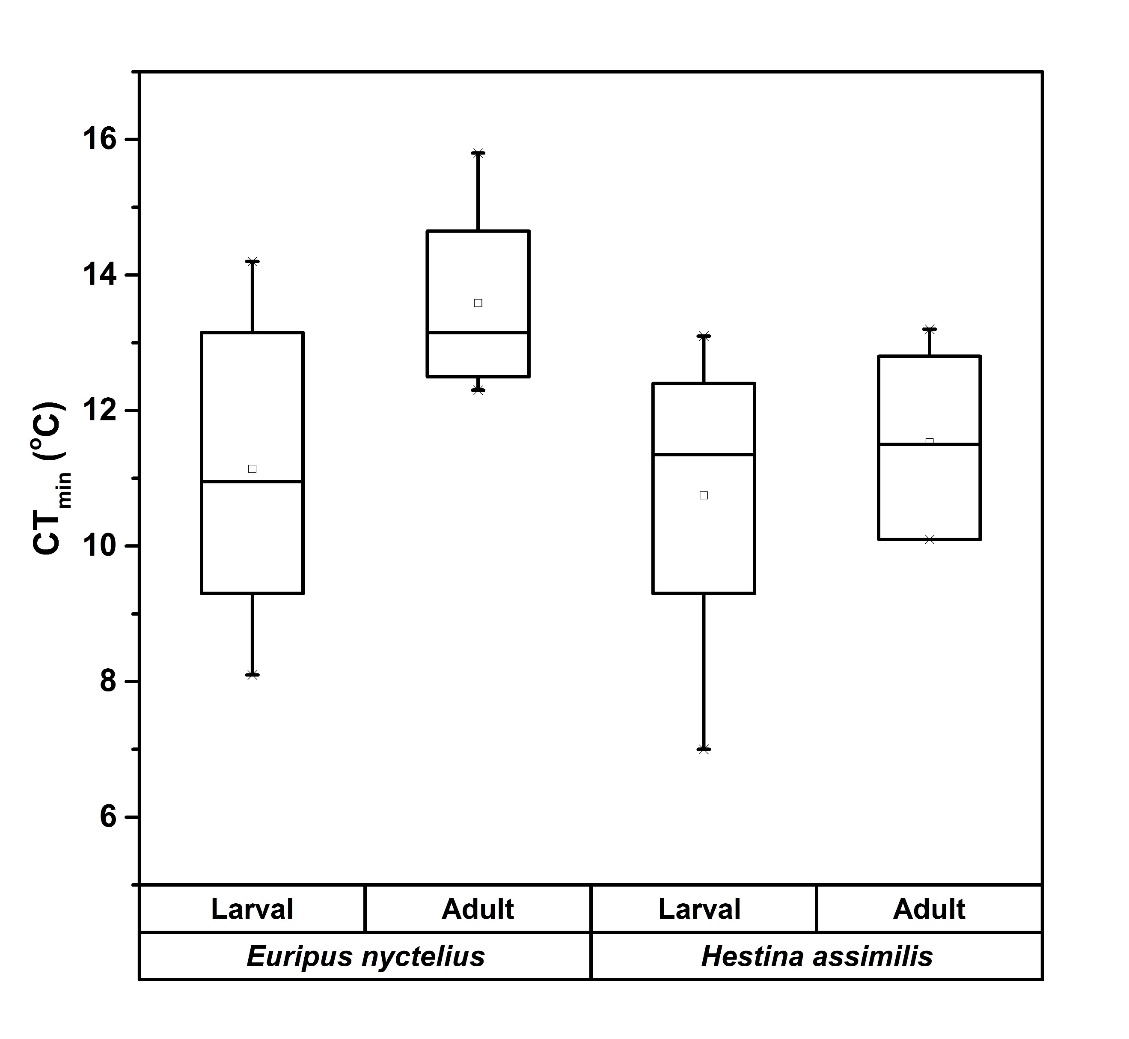


**Supp. Figure S2.** Critical thermal minimum (CT_min_) of the same *Euripus nyctelius* and *Hestina assimilis* larva and adult individuals which survived both stages.

**Supp. Table S1.** The sources, coordinates and locations of occurrence records employed in species distribution models for *Euripus nyctelius* and *Hestina assimilis* (Refer to excel file)

**Supp. Table S2.** Bioclimatic variables chosen for ecological niche modelling

| Abbreviation | Parameter |
| --- | --- |
| BIO2 | Mean Diurnal Range (Mean of monthly (max temp - min temp)) |
| BIO4 | Temperature Seasonality (standard deviation × 100) [°C] |
| BIO8 | Mean Temperature of Wettest Quarter [°C] |
| BIO10 | Mean Temperature of Warmest Quarter [°C] |
| BIO13 | Precipitation of Wettest Month [mm] |
| BIO14 | Precipitation of Driest Month [mm] |
| BIO15 | Precipitation Seasonality (Coefficient of Variation) [%] |
| BIO18 | Precipitation of Warmest Quarter [mm] |
| BIO19 | Precipitation of Coldest Quarter [mm] |
